# Developing a coordinated response to chemsex across health, justice and social care settings: expert consensus statement

**DOI:** 10.1192/bjb.2024.46

**Published:** 2024-10

**Authors:** Bradley Hillier, Eliott Carthy, Nicola Kalk, Monty Moncrieff, Mark Pakianathan, Derek Tracy, Owen Bowden-Jones, Ford Hickson, Andrew Forrester

**Affiliations:** 1 West London NHS Trust, London, UK; 2 London School of Hygiene and Tropical Medicine, London, UK; 3 University of Oxford, Oxford, UK; 4 South London and Maudsley NHS Foundation Trust, London, UK; 5 Institute of Psychiatry, Psychology & Neuroscience, London, UK; 6 London Friend, London, UK; 7 Guy's & St Thomas’ NHS Foundation Trust, London, UK; 8 Central and North West London NHS Foundation Trust, London, UK; 9 Cardiff University, Cardiff, UK

**Keywords:** LGBTQ+, stigma and discrimination, substance use disorders, chemsex behaviour, criminal justice system

## Abstract

Chemsex occurs primarily among gay, bisexual and other men who have sex with men (GBMSM), and there is evidence of a subgroup of users who carry out chemsex-related criminal offences and experience harm. Challenges with chemsex can present to various settings; there are concerns that harm is increasing, including at interfaces between health, social care and criminal justice systems. The UK response to date has lacked a coordinated approach. An expert reference group was convened to share chemsex knowledge, articulate priorities for research and pathway development, and foster collaborative working between agencies. It made three key recommendations: develop and increase training and awareness across all services; implement a coordinated research programme with the development of a common data-set and assessment tool to fully characterise population-level needs; develop a professional network to share information, provide professional support and act as a knowledge hub. There was support for a unified multi-agency strategy incorporating the priorities identified as overarching principles.

Chemsex refers to the use of specific substances before or during sex to sustain, enhance, disinhibit or facilitate the sexual experience, primarily among gay, bisexual and other men who have sex with men (GBMSM).^1^ Although there are a variety of conceptual definitions, the primary drugs associated with chemsex in the UK are crystal methamphetamine (‘crystal’, ‘Tina’, ‘T’), gamma-hydroxybutyrate/gamma-butyrolactone (GHB/GBL – ‘G’), mephedrone and ketamine, although other types may also be involved.^1–3^ These drugs are often taken in combination, with or without additional psychoactive substances such as ecstasy, cocaine and/or non-psychoactive substances such as sildenafil and alkyl nitrites. It is typically facilitated by location-based social networking apps (e.g. Grindr, Scruff) and may involve online contact, multiple partners and prolonged periods of sexual activity over several days.

There are complex biological, psychological and social factors that influence why someone may choose to engage in chemsex that are yet to be fully understood.^4,5^ The prevalence of chemsex also likely varies between countries and between studies, underpinned by factors such as under-recognition and variable definitions of the term.^6^ It is recognised that although it is a minority practice among a minority population, it is disproportionately associated with significant complex harms across a number of health, social care and criminal justice domains (e.g.^4,6^). Examples include increased risk of HIV and other sexually transmitted infections, harms from direct intoxication and overdose of the substances involved, and an increasing cohort of individuals in prison in the UK owing to violent offending either associated with, or contextualised within, chemsex (Stephen Morris, personal communication, 2023). Furthermore, although chemsex has been recognised as an area of clinical and societal need by various UK agencies (e.g. the Advisory Council for the Misuse of Drugs (ACMD), the National Health Service (NHS), voluntary sector health services, academics and the justice sector) there are currently none of the following in existence:

- a universally agreed definition
- a national UK chemsex strategy
- a national service specification
- nationally or internationally recognised standards of care or treatment
- a standardised assessment process
- a specific rating scale.

There is also a significant lack of resources and research evidence to inform these. Notwithstanding this, there are recognised ‘pockets of excellence’, existing independently in places where there are larger GBMSM populations (e.g. the Antidote service: www.londonfriend.org.uk), usually having developed in response to local needs, following the interest and engagement of local experts.

The need to develop and coordinate a national integrated response to chemsex occurs alongside increasing international recognition of the challenge, as well as the close association that exists with new HIV infection and the UNAIDS strategic target to end the AIDS pandemic by 2030.^7^ As regards chemsex in particular, this aligns with four of the strategy's Sustainable Development Goals (SDGs): good health and well-being (SDG 3), reduced inequalities (SDG 10), peace, justice and strong institutions (SDG 16) and partnerships for the goals (SDG 17).^7^

In 2020, the Spanish Ministry of Health published a technical document to better understand the prevalence and patterns of chemsex in Spain and made recommendations on how to address the health-related harms associated with it at both a local and national level.^8^ In England, the government published its LGBT Action Plan (2018),^9^ although it does not make specific mention of chemsex, and Public Health England (2015) published a guide to raise awareness of substance use services for men involved in chemsex,^10^ but no overarching strategy is included. Part 2 of the Independent Report into the Review of Drugs by Dame Carol Black acknowledged that ‘some very vulnerable groups, such as [ … ] people who use image- or performance-enhancing drugs or people engaged in “chemsex”, do not receive an adequate or any service, but are at great risk’ (para. 3.2).^11^ In short, recognition is occurring, but the response remains limited, uncoordinated and highly variable; this was also the only use of the term ‘chemsex’ in either of the two parts of the report, which, given the awareness of this practice in the GBMSM community, raises concerns as to whether there has been sufficient engagement with the issue and how appropriate services might be developed.

## Chemsex Expert Reference Group

The Chemsex Expert Reference Group (ERG) is a group of NHS, independent and voluntary sector clinicians, academics, and public health and justice professionals with experience and expertise in working with chemsex and its associated challenges across multiple settings. It aims to improve understanding of the health-related harms that can be associated with chemsex in the UK and how to address these in a coordinated evidence-based manner. This consensus statement summarises the outcomes of the first national meeting of the Chemsex ERG in London in summer 2022, attended by national experts from across the UK. The meeting took place at the London School of Hygiene and Tropical Medicine (LSHTM) and was co-hosted by West London NHS Trust.

The provisional aims of the meeting were to understand the current experiences in various professional settings of the health and social harms that can be associated with problematic chemsex and the experiences of professionals working with chemsex users through multiple different agencies, and was guided by three overarching questions for attendees to consider in advance of the meeting:

- What do we need to know about working with this complex and vulnerable group of people?
- What is the research needed to improve our understanding?
- What are the aspirational clinical pathways that should be developed?

## Method

The meeting's organisers and their affiliations are listed in the authorship of this position statement. Table 1 summarises the breadth of expertise of those who attended and participated in the Chemsex ERG. The day consisted of four main sessions, focused on setting the scene, sharing knowledge about the problem from different sector perspectives, discussing solutions and, finally, priority/agenda setting for future work.

**Table 1:** Representation of Chemsex Expert Reference Group participants

Healthcare	Voluntary sector	Criminal justice	Academia
Adult psychiatry (addiction, forensic, liaison, homeless) Emergency medicine Ambulance services Sexual health and HIV medicine General practice Public health	Addiction services (Antidote, Forward Trust, Turning Point) MEN R US (civil society organisation)	London Metropolitan Police Service (MPS) His Majesty's Prison and Probation Service (HMPPS) Court liaison and diversion services (provided by health and third sector)	London School of Hygiene and Tropical Medicine Cardiff University University College London Imperial College London King's College London

A scene-setting plenary session heard from the perspective of public health (F.H.), the ACMD work on GHB/GBL and future directions in LGBTGI+ (O.B.-J.) and third-sector interventions (M.M.). This was followed by a ‘round robin’ session consisting of 15 brief (7 min) presentations from the diverse group of attendees summarising their roles, experiences of working with people engaging in problematic chemsex within their particular service/sector/interface, and the three main challenges they faced and gaps in knowledge and resources they saw as priorities.

The third session identified two evenly balanced breakout groups with a cross-section of representatives from different sectors, who contributed to a 75 min facilitated discussion focusing on the three main questions of the day as detailed above. The final session included structured group feedback (facilitated by D.T., B.H. and E.C.) and prioritisation of identified needs requiring further action.

A Member of Parliament with a strong interest in supporting recovery from addiction also contributed for part of the day.

## Results and key themes

The group identified key points and actions related to the three main questions outlined above. Although the points and approaches identified are likely to overlap with those used in other countries (such as those highlighted by the Spanish Ministry of Health,^8^ such as the need for multi-agency collaboration), the practicalities of how this can be implemented will vary between countries and healthcare systems. This merits country-specific research and the development of a national UK-wide approach to chemsex that is able to draw on developments and initiatives elsewhere.

### Objective 1: What do we need to know about this population?

The ERG identified several gaps in our current knowledge and evidence base. These can be grouped into the following areas:

- a lack of epidemiological data
- the importance of cultural competency regarding the health needs of LGBTQ+ people, especially in identifying when presentations may be related to chemsex
- uncertainties about the knowledge of healthcare professionals on how to manage emergency presentations such as methamphetamine-induced psychosis, GHB withdrawal and GHB overdose, and risk assessment
- risk management for those who may also be a victim and/or a perpetrator of a criminal offence in a chemsex context.

#### Identification and assessment

There is a lack of data on the prevalence of chemsex in the UK and how the harms that can be associated with it present to clinical services. People may engage in chemsex for multiple and complex reasons, and may present to a range of different clinical and non-clinical services, which, in turn, can influence long-term support for better or worse. For example, the anecdotal experience of attendees at the meeting was that people engaging in chemsex are more likely to come to the attention of community sexual health services than community health or drug and alcohol services. This may be influenced by their specific health need at the time, but may also be owing to past experience of how knowledgeable professionals were or perceived cultural competency around LGBTQ+ issues that mean individuals felt understood and supported rather than judged or stigmatised.^10^

The ERG described the challenges of recognising when someone's presentation may be in the chemsex context, regardless of the setting in which it presents. Many attendees shared their experiences of feeling disconnected from their colleagues with regard to understanding of chemsex, as well as feeling helpless to identify what acute and longer-term support is available. This was suggested to arise from a combination of stigma towards the chemsex group, services being overwhelmed by other more ‘deserving’ challenges, and a genuine lack of awareness/understanding of the problem. Many recognised that each clinical contact was a potential ‘reachable moment’, because if the clinician with even a minimal understanding of chemsex had contact with a chemsex user, there was the potential to engage in a meaningful way, rather than missing an opportunity. Professionals wished they had more practical solutions and support options available to promote at these times.

#### Cultural competency on LGBTQ+ health (a strong theme across sectors)

The ERG considered that the need for professionals to have good competency in diversity issues and chemsex was of vital importance. There is substantial evidence of an association between being LGBTQ+ and poorer mental health.^12^ In addition, qualitative interviews have identified that people engaged with chemsex are commonly concerned about additional stigma from support services if seeking help.^13^ These are indicators of a potentially higher burden of mental illness and reduced access to support for those engaging in chemsex in addition to that already experienced by LGBTQ+ people.

The ERG shared experiences of how feelings of stigma and/or shame may be a factor in why people are more likely to present through sexual health services than through community drug and alcohol services; it was acknowledged that sexual health services are more readily accessed by LGBTQ+ people rather than community drug and alcohol services, although such services may not themselves feel well equipped to manage addiction, psychiatric and psychosocial chemsex problems beyond the sexual health implications. Feeling understood and supported is key to therapeutic engagement for a group who may have also experienced varying forms of trauma, which was another need identified in understanding the population.

LGBTQ+ people have reported experiences with mental health services that reinforce stigma and display a lack of understanding of their specific needs.^14^ Similarly, a survey of users of the London-based LGBTQ+-focused drug and alcohol service Antidote found that many users preferred to access an LGBTQ+-specific service and were more comfortable disclosing and discussing their sexuality in this setting (Monty Moncrieff, personal communication, 2023); some felt that there was more interest in discussing the background and reasons for their drug use than in mainstream services. Previous bad experiences with stigma and/or lack of understanding of their lifestyle from mainstream services had led to some users not disclosing their sexuality at all to mainstream services.

An associated aspect of vulnerability that should be considered within LGBTQ+ competency is the potential for those involved in chemsex to be subject to modern slavery in the context of both sex work and non-sex work. Regardless of the setting in which the individual presents, a core competency of any framework would need to involve an LGBTQ+-sensitive approach to safeguarding informed by minority stress theory, as well as familiarity with the National Referral Mechanism for reporting modern slavery.

#### Emergency care and mental health

Chemsex can present multiple challenges to emergency workers, such as those in the ambulance service and emergency departments. Anecdotally, some patients are concerned that by calling the emergency services for a medical emergency they may be subsequently arrested for drug-related offences. An unconscious patient (e.g. after GHB overdose) may require complex medical management such as airway support, and chemsex parties may mean there are multiple people in need of treatment. Professionals reported having little to no information about the individual's medical history, allergies or what substances they have taken, and limited awareness of the risks involved. There may also be safeguarding and/or criminal concerns identified, for example regarding consent to sexual activity, exploitation in exchange for substance use, more serious violence (such as varying degrees of physical and sexual assault and rape) and a dual victim/perpetrator identity.

Training and knowledge of how to manage GHB overdoses was noted to vary widely, and emergency staff in areas where chemsex is less common may struggle to manage these situations, since there is no formalised or mandatory training. Similarly, there are marked difficulties and delays in access to specialist community and in-patient services for GHB detoxification where needed. Although some community services provide ambulatory GHB detoxification, others do not, and in-patient detoxification can involve a delay of months.

Methamphetamine use has been associated with a five-fold increased risk of psychotic symptoms, that increases with duration and frequency of use and with concomitant use of alcohol and cannabis.^15^ Although most episodes of psychosis resolve within a few weeks of abstinence, approximately 30% last longer than a month.^15^ The symptomatology of methamphetamine-induced psychosis overlaps with that of schizophrenia^15^ and may become persistent.^16^ Individuals may therefore be referred to secondary care mental health services, including to community mental health teams, early intervention services and liaison psychiatry, from the emergency department or from police emergency holding. They may therefore also be admitted to in-patient psychiatric wards under mental health legislation and be prescribed antipsychotic treatment. The natural history of methamphetamine-induced psychosis remains unclear and complex, and it is not straightforward to identify who may be at risk of developing a persistent psychotic disorder and their risk factors for this, or the way that treatment should occur in the emergency setting. In other cases, patients who suffer from persistent psychotic symptoms are turned away from secondary mental healthcare as their presentation is attributed to drugs and seen by mental health professionals as the remit of community addiction services, which are often neither resourced nor skilled to provide assessment and management of a psychotic illness.

#### Criminal offending

The potential association between chemsex, mental illness and criminal offending remains largely unstudied, although there is an increasing awareness of a relationship to offending behaviours. In response to some of the high-profile criminal cases involving serious harm (for example, that of Stephen Port, who murdered four young men in London using GHB overdose) and in recognition of the rising number of offences occurring within a chemsex context, the Metropolitan Police Service and His Majesty's Prison and Probation Service (HMPPS) set up Project Sagamore to improve the identification and management of such cases (Allen Davis, personal communication, 2023). Recently published data from Project Sagamore used a criminal justice definition developed in HMPPS to identify such cases when they arose, and the HMPPS chemsex lead reported 256 active chemsex-related convictions in London, which mainly include crimes of physical and/or sexual violence and high rates of re-offending in the chemsex context on release from prison.^17^ This is likely to be a highly selected sample, as it was identified anecdotally without systematic process, and indicative of the more severe end of the chemsex and offending spectrum, and therefore limited in terms of the conclusions that can be drawn from it; nonetheless, similarly to the under-recognition of chemsex in health settings, offending is likely to be under-recognised as being in a chemsex context according to this report.^17^

### Objective 2: What are the research priorities?

The ERG considered that there were significant needs in terms of both utilising and adapting existing data collection tools, as well as the necessity to develop new, research-informed chemsex-specific ones.

The limited data concerning the prevalence of chemsex, inconsistent/absent recording and coding (including the existence of any code at all), and the personal and health demographics of those engaging in chemsex, particularly those who come to harm in some way, was the first key research priority identified by the ERG. Although some pockets of data (such as through the Genito-Urinary Medicine Clinic Activity Dataset (GUMCAD) or Treatment Outcomes Profile (TOP)) may contain some useful information, they are not mandatory, nor do they reflect the complexity in which chemsex interfaces with the health, social care and criminal justice systems.

Examples of how existing tools/data-sets could be optimised include:

- provision of funding for sexual health services to complete the current GUMCAD (GUMCAD v3), and making GUMCAD data capture and reporting mandatory for use of chemsex drugs;^18^
- optimising data in the National Drug Treatment Monitoring System (NDTMS; www.ndtms.net):
  - reach a target 95% completion of the sexual orientation field
  - publish data annually relating to methamphetamine, ketamine, mephedrone, and GHB, GBL and related substances (GHBRS) by sexual orientation;
- including GHBRS use and sexual orientation in the Crime Survey of England and Wales (www.crimesurvey.co.uk);
- providing funding for the Gay Men's Sex Survey (part of the European MSM Internet Survey – EMIS) for the next 5 years (previously this was funded by the European Union);
- involving pathology and toxicology services, providing funding for mandatory testing for GHB in all unexplained deaths (taking into account its complex pharmacokinetics):^19^
  - studies such as IONA (Identification of Novel Psychoactive Substances) in patients seeking emergency care will also provide useful data linked to clinical information, potentially allowing earlier identification of novel chemsex drug use.^20^

Through the optimisation of these tools in a coordinated way using an agreed terminology and question format it may be possible to extract comparable data relating to chemsex for analysis within different (but related) populations. Data from such a process could be valuably used to inform service needs, development and associated funding requirements, as well as to guide further research in an organised way.

#### Developing existing chemsex research

The group considered that there was an urgent need to articulate and attempt coordination of chemsex research activity in the UK, building on current research expertise, which has been growing over the past 10–15 years in the UK and internationally.

A key challenge has been that there is no consistently identified definition for ‘chemsex’ in research methodology, so a variety of closely related but ultimately differing terms have been developed. Following from this, both quantitative and qualitative research methods are needed to understand in more detail:

- the prevalence of chemsex (and associated harms) in a variety of different settings
- the relative contribution of chemsex within these cases – e.g. primary contribution, major contribution, minor contribution, association, etc.
- what services and knowledge currently exist (e.g. surveys of existing teams and individuals to assess their knowledge and service bases)
- the effectiveness of training in this area through training programme implementation and evaluation
- screening and identification of chemsex in different settings, including how best to do this, for example along with existing screens or separately when a concern appears
- the complexity of individual cases – e.g. following through a number of cases from start to finish to assay their complex journeys and the interactions with professionals, services, systems, etc.
- additional questions regarding crime-related aspects in studies such as EMIS
- how chemsex users may differ from others and their priorities – case–control studies could be useful for this
- support pathways – setting up pilot pathways across agencies to understand whether they can be effective in supporting people into care/treatment/accommodation, etc.
- the ways in which people engage in harm minimisation, risk factors leading to addiction, and how recovery is achieved and sustained.

The above describes an ideal, overarching and highly ambitious research agenda that would take significant time and resources to deliver and would be very difficult to begin from the current state of knowledge, albeit that it is aspirational to fully characterise the phenomenon. A more realistic series of stages, some of which are already in progress, would be as follows:

- stage 1: systematic survey and qualitative work to characterise chemsex behaviour in different populations (currently occurring *ad hoc*)
- stage 2: prevalence work in sexual health clinics and other settings (e.g. emergency departments, justice settings, addiction services – currently occurring *ad hoc*)
- stage 3: pilot screening to deliver a screening tool to help identification (there is limited evidence that this is occurring)
- stage 4: pilot management/treatment models (there is some evidence of this occurring but in a non-coordinated way on a basis of limited evidence).

A further, associated, research priority would also include the ongoing development of an LGBTQ+ specialism and/or accreditation within training programmes, based on research and an understanding of minority stress theory; this may also help meet the objectives of the LGBT Action Plan (2018).^9^

The ERG acknowledged the anecdotal experience of healthcare professionals in services that have been successful at engaging with chemsex users as being vital for this, as well as promoting the importance of lived experience. It was also recognised that there continues to be uncertainty as to how best to engage with people with lived experience in a manner that is health-promoting, without fear of discrimination or that seeking help and support may risk criminal prosecution.

### Objective 3: What are the aspirational clinical pathways we want to create?

The ERG identified authenticity, competence and a non-judgemental approach as core values for service and pathway development and felt that lived experience, carer and family engagement and high-quality research should be at the centre of service development. Interventions for assessment and treatment should be evidence based and supported by national clinical guidelines that not only balance individual needs with wider population needs, but also provide a set of agreed standards that can be audited against; safeguarding should form a basis of any pathway.

Multiple different areas of harm have been associated with chemsex, across physical, sexual and mental health services, emergency services (including police and ambulance), the criminal justice service, and housing and homelessness agencies.^13,21^ Currently, care is delivered to individuals, but there are also population-level considerations and the reality of multiple services and competing needs. Different services often work in silos and are unable to, or find it time-consuming to, share data; for example, some organisations accept only self-referrals, not allowing referrals from professionals – this can make information sharing difficult if a professional is the individual's first point of contact. Such situations may structurally disadvantage minority populations and lead to people becoming lost in the system. There is a need for sustained pressure on policymakers to review commissioning arrangements to facilitate access to health services, and a drive to ensure that health and criminal justice considerations are balanced. This is beginning to happen through the Independent Review by Dame Carol Black^11^ and through recommendations from the ACMD (e.g. in relation to GHBRS),^22^ although there is still significant progress to be made, as detailed above.

Collaborative working across sectors and agencies is needed for research and service development. In particular, better integration is needed between mental health, substance use and sexual health services, but police, prison and national crime agencies also should work together and with other agencies, including relevant health services. Psychiatry, mental health and public health should be key partners in the development of evidentially informed clinical pathways, as well as working with the criminal justice system and other services to develop interventions that reduce risk and recidivism and improve health for offenders while safeguarding the public. Cross-boundary working (including with criminal justice agencies) and collaboration should include an agreed data-sharing protocol, which may be facilitated by the development of a common baseline assessment for multiple services to capture and record data. This should include mandatory sections focusing on sexual and mental health, substance use screening and markers of social vulnerability (which should include elements such as isolation, intersectional disadvantage and identity secrecy). Owing to the sensitive nature of the information there would need to be very robust assurances concerning data security and access and clear delineation of health versus justice data-sets, while recognising the mutual benefit of high-level information sharing in both the detection and prevention of crime, and the need for data from diverse non-health settings.

One example of how multi-agency working has developed to encompass the broad range of agencies encountering specific challenges is the multi-agency public protection arrangements (also known as MAPPA). These are very specifically justice-led on the basis of risk that may be posed by individuals and how various agencies can work together to share information (where appropriate) and identify and manage risks in different domains.^23^ A similar information-sharing structure between police and mental health agencies has developed through the Fixated Threat Assessment Centre (FTAC),^24^ arising from a research project on the development of a clinical service. There may be other examples which, through research, can inform the most appropriate approach in each sector.

A difficult area to address is commissioning bias due to the presence or absence of data, as is the need for efforts to be made to generate relevant data to argue for a service that in turn collects activity data and informs commissioning; this may also be improved by effective collaboration and multi-agency working, with the sharing of high-level population data described earlier to obtain a clearer picture; in this sense the absence of evidence does not mean evidence of absence. Chemsex is a minority practice and is typically most common in urban areas, which also makes it more likely that staff in areas with low numbers of chemsex users will lack training and knowledge. It is impractical to provide specific services for chemsex users everywhere, but it may be possible to extend the reach of existing services by adding an LGBTQ+ specialism as part of the research agenda described above. Regional hubs, or even a national training hub with specialist trained staff in LGBTQ+ care whose remit it is to train existing services, may be a practical option, building on existing services, which are primarily within regional cities with large LGBTQ+ populations (e.g. Manchester, Brighton) and London, in conjunction with a mapping process; developing partnerships between local and regional/national organisations may also be an option. Online services may be available in some cases, although again individuals’ access preferences should be considered.

The ERG considered following characteristics valuable in the development of clinical pathways.

- Regular staff education and training on chemsex, including sensitivity training, to increase the number of staff familiar with the challenges who actively seek to reduce stigma and trauma, initially through a pilot and with a view to integrating into mandatory training. This should include:
  - specialist training of front-line and emergency services staff on GHBRS, and coverage of these drugs in training curricula
  - engagement with front-line policing to inform the identification of chemsex-involved individuals at the initial point of contact and/or arrest, with a view to ensuring that this is addressed clinically, safeguarding approaches are activated and data are captured
  - information about acute health emergencies associated with chemsex, e.g. strokes, heart attacks, rhabdomyolysis and renal failure, psychosis associated with methamphetamine, and GHBRS overdose and withdrawal, with the potential complications of seizures and delirium
  - information about chronic harms and the context of chemsex (trauma and stigma), and non-acute presentations, e.g. elective GHB detoxification
  - use of bite-sized teaching (BST) and regularly updated, peer-reviewed online material; voluntary sector organisations may be helpful
  - bodies such as Health Education England, NHS England, the Academy of Medical Royal Colleges, British Association for Sexual Health and HIV (BASHH), UK Health Security Agency (UKHSA) and associated bodies in the devolved nations.
- Provision of clear and helpful information to service users in various formats – this could include a card with a QR code to scan for further information, such as on the Gay Men's Health Collective website (GMHC; gaymenshealthcollective.co.uk):
  - information should address fears about encounters with the police if calling for medical help: current packs from the GMHC include information about chemsex, calling an ambulance and rights on arrest; these packs are provided currently in some emergency departments, and this could be rolled out further
  - information packs could be provided at ‘reachable moments’, such as after seeing someone else have an overdose
  - an online self-assessment tool with further links could help men to understand their chemsex use and access information and help.
- Where possible, research into whether the introduction of health navigators to guide service users through pathways would be beneficial within chemsex, as is recognised more generally for those who have significant health complexity.
- Education and better understanding of chemsex on the part of those involved in sentencing decisions, including mental health-related disposals; this may form a part of a wider programme of delivering training to a range of staff, including psychiatry, probation services and the judiciary, as well as part of existing training.

#### Next steps for the Chemsex ERG

The following were agreed as next steps for the ERG. The consensus was to capitalise on this meeting by the production of the position statement, by formalising the group, and by enabling further work on key research priorities and taking the following steps towards facilitating multi-agency collaboration:

- formalising a structure for the group, including accountability and governance
- developing a parliamentary group, for example LGBTQ+ focused, relating to chemsex, addiction, and mental and physical health
- supporting the recommendations of the ACMD report on GHBRS^22^
- collaborating to call for a national strategy specifically for addressing chemsex and associated health and justice disparities
- facilitating professional network groups to promote best practice and guidelines
- articulating the need for a specific research programme with national scope
- developing networks with international partners to share learning and best practice and collaborate in research
- developing and funding national and international conferences in this area.

## Conclusion

The chemsex ERG is, as far as we are aware, the first truly high-level, multi-agency and multi-sector event to be held in the UK aiming to inform a coordinated approach to a health and evidence-based response to chemsex. The event helped identify a range of important and achievable goals to improve the way that the health harms associated with chemsex can be addressed on an individual and population level. The ambition, interest and engagement from across many diverse clinical specialties, third-sector organisations, academia and the criminal justice system highlighted the appetite to develop and articulate a comprehensive response to chemsex and the challenges and vulnerabilities both leading to and arising from it. It also helped to share positive work already occurring within sectors and facilitate networking and relationships from a broad group of representatives.

The ERG has been very much a first step towards the clear need for a formalised and professionalised group that can advocate for the necessary coordination to develop such an approach. It is very clear that a major absence in the literature and in government policy is any form of health or multi-agency strategy that attempts to address at least some of the challenges identified in this document, and we would call for this to be a health priority. Such a strategy will only be useful if it engages with chemsex users and their carers (as well as experts and current services) and settings not traditionally associated with health matters (such as the within the criminal justice and housing systems).

Although data remain limited, there is good evidence that in other countries (e.g. Spain and France) this has been recognised as a priority on the grounds of equity, and the ACMD made chemsex a priority several years ago. The development of the response to date in the UK has been organic and driven by passionate individuals with a strong personal interest, frequently from withing the GBMSM community, taking an advocacy approach; inevitably, such an approach will provide excellence in some areas and variability elsewhere, given the absence of a widely agreed and shared set of service standards. Such variability across the UK contributes to health inequalities within an already stigmatised group (GBMSM) engaged in stigmatised activities (chemsex; other drug use) and among whom there is association with another stigmatised health problem (HIV). In the opinion of the ERG, this multiple stigmatisation makes a strong argument for an urgent need to invest the necessary resources not only in existing services, but in the priorities identified here, underpinned by a national strategy. We hope that the members of the ERG and this consensus statement can help progress to a more cohesive development of an evidence-based, national, multi-agency approach to research and understanding, engagement, assessment, treatment, pathway development and recovery for a group of individuals with complex needs.

## Data Availability

Data availability is not applicable to this article as no new data were created or analysed in this study.

## About the authors

**Bradley Hillier** is Chair of the Health and Justice Liaison Group to Project Sagamore (London Metropolitan Police and His Majesty's Prison and Probation Service (HMPPS), London, UK), a consultant forensic psychiatrist with West London NHS Trust, London, UK, and the Health and Community Services Department in Jersey, Channel Islands, and has submitted his dissertation for a Masters in Public Health at the London School of Hygiene and Tropical Medicine, London, UK. **Elliott Carthy** is an honorary specialist registrar in forensic psychiatry with West London NHS Trust, London, UK and a lecturer in medicine in the Department of Psychiatry, University of Oxford, Oxford, UK. **Nicola Kalk** is a consultant addiction psychiatrist with South London and Maudsley NHS Foundation Trust, London, UK, and a senior lecturer in the Department of Addictions, Institute of Psychiatry, Psychology & Neuroscience, King's College London, London, UK. **Monty Moncrieff** is Chief Executive of London Friend, providing Antidote's specialist chemsex recovery service, London, UK. **Mark Pakianathan** is a consultant physician in sexual health and HIV medicine with Guy's & St Thomas’ NHS Foundation Trust, London, UK, and Deputy Chair of the Health and Justice Liaison Group to Project Sagamore, London, UK. **Derek Tracy** is a consultant psychiatrist and medical director of West London NHS Trust, London, UK. **Owen Bowden-Jones** is a consultant addiction psychiatrist with Central and North West London NHS Foundation Trust, London, UK, and Chair of the Advisory Council on the Misuse of Drugs, London, UK. **Ford Hickson** is an associate professor and programme director for the MSc in Public Health at the London School of Hygiene and Tropical Medicine, London, UK. **Andrew Forrester** is Professor of Forensic Psychiatry in the Department of Psychological Medicine and Clinical Neurosciences, School of Medicine, Cardiff University, Cardiff, UK.
